# Visually guided classification trees for analyzing chronic patients

**DOI:** 10.1186/s12859-020-3359-3

**Published:** 2020-03-11

**Authors:** Cristina Soguero-Ruiz, Inmaculada Mora-Jiménez, Miguel A. Mohedano-Munoz, Manuel Rubio-Sanchez, Pablo de Miguel-Bohoyo, Alberto Sanchez

**Affiliations:** 1 Department of Signal Theory and Communications, Telematics and Computing Systems, Rey Juan Carlos University, Fuenlabrada, Spain; 2 Department of Computer Science & Statistics, Rey Juan Carlos University, Fuenlabrada, Spain; 3 University Hospital of Fuenlabrada, Fuenlabrada, Spain; 4 Research Center for Computational Simulation, Montegancedo, Spain

**Keywords:** Chronic health status, Diabetes, Hypertension, Multivariate visualization, Classification trees, Diagnoses, Drugs

## Abstract

**Background:**

Chronic diseases are becoming more widespread each year in developed countries, mainly due to increasing life expectancy. Among them, diabetes mellitus (DM) and essential hypertension (EH) are two of the most prevalent ones. Furthermore, they can be the onset of other chronic conditions such as kidney or obstructive pulmonary diseases. The need to comprehend the factors related to such complex diseases motivates the development of interpretative and visual analysis methods, such as classification trees, which not only provide predictive models for diagnosing patients, but can also help to discover new clinical insights.

**Results:**

In this paper, we analyzed healthy and chronic (diabetic, hypertensive) patients associated with the University Hospital of Fuenlabrada in Spain. Each patient was classified into a single health status according to clinical risk groups (CRGs). The CRGs characterize a patient through features such as age, gender, diagnosis codes, and drug codes. Based on these features and the CRGs, we have designed classification trees to determine the most discriminative decision features among different health statuses. In particular, we propose to make use of statistical data visualizations to guide the selection of features in each node when constructing a tree. We created several classification trees to distinguish among patients with different health statuses. We analyzed their performance in terms of classification accuracy, and drew clinical conclusions regarding the decision features considered in each tree. As expected, healthy patients and patients with a single chronic condition were better classified than patients with comorbidities. The constructed classification trees also show that the use of antipsychotics and the diagnosis of chronic airway obstruction are relevant for classifying patients with more than one chronic condition, in conjunction with the usual DM and/or EH diagnoses.

**Conclusions:**

We propose a methodology for constructing classification trees in a visually guided manner. The approach allows clinicians to progressively select the decision features at each of the tree nodes. The process is guided by exploratory data analysis visualizations, which may provide new insights and unexpected clinical information.

## Background

The increase in life expectancy in developed countries is originating multiple chronic conditions, which may appear both independently and jointly in individuals. Their analysis is important for several reasons [1]: (1) they account for 60% of global deaths; and (2) they trigger 75% of public health expenditure (for instance, 80% of primary care consultations, and 60% of hospital admissions, are related to chronic diseases).

Essential hypertension (EH) and diabetes mellitus (DM) are among the most common chronic conditions nowadays. Furthermore, it is expected that the number of patients suffering from these chronic conditions will increase, reaching the figure of 1.56 billion hypertensive patients in 2025 [2], and 552 million diabetic patients in 2030 [3]. In addition, EH and DM frequently occur together. For example, in the US population, EH is present in approximately 30% of patients with type 1 diabetes, and in 50–80% of patients with type 2 diabetes [4, 5]. Furthermore, the consumption of antihypertensive drugs may increase the probability of suffering from DM [6].

There are several strategies in the literature for identifying patients with chronic conditions. For example, Smith et al. [7] uses questionnaires, while [8, 9] are based on electronic health records (EHR). In the latter, clinical population groupers take into account data from the EHR in a specific period of time, and assign patients to a unique and mutually-exclusive health status. The population classification system called Clinical Risk Groups (CRGs) [10, 11] distinguishes more than one thousand health statuses (groups) and is orientated towards categorizing chronic patients.

In this work we consider the CRGs system not only because it has been internationally validated, but also since it defines groups that include patients with several chronic conditions (comorbidities). Specifically, the CRGs system uses demographic data (age and gender), as well as information about diagnoses, procedures, and drugs, in order to determine a patient’s health status during a period of time (typically, one year).

The stratification provided by the CRGs system has been used in several works to create non-parametric models for classification through statistical learning techniques. Fernández-Sánchez et al. [12] use age, gender and diagnosis codes in order to classify patients into three health statuses (healthy, hypertensive, and hypertensive with comorbidities) by using Support Vector Machines (SVMs). Since the models provided by SVMs can be considered as “black boxes”, it can be difficult to extract clinical knowledge from them. Therefore, Soguero et al. proposed the use of classification trees (also called decision trees), which are statistical models that are easy to interpret [13]. The particular approach was based on the C4.5 algorithm [14], which is one of the simplest methods for constructing classification trees automatically (with no human supervision). Additionally, the work in [13] extends the analysis in [12] by considering larger sets of features and chronic conditions. In particular, it evaluated four kinds of features (age, gender, diagnosis codes, and drug codes), and two chronic conditions (EH and DM), also considering comorbidities with other chronic diseases.

In this paper we extend the analysis of classification trees in [13] by allowing clinicians to select the decision features (i.e., the ones used to partition the dataset) at each of the nodes of a tree. In particular, we propose using data visualizations to guide the decision feature selection process when constructing the trees, and where clinicians can rely on their domain knowledge when selecting the decision features.

Specifically, the process of choosing the most discriminative features at each level of the tree is guided by star coordinates (SC) plots [15, 16]. SC is a multivariate visualization method based on radial axes [17–19] that produces linear projections of data samples (which are usually depicted as dots). In this work, every sample corresponds to one patient, who is represented by a high-dimensional data point mainly composed of clinical codes. In addition, the approach also shows a set of two-dimensional vectors graphically, each associated with one feature. This set of vectors specifies a particular linear projection that maps high-dimensional data points onto a plane. Since SC can be coupled with any linear projection method (see [20–22]), in this paper we consider a combination of SC with linear discriminant analysis (LDA) [23]. LDA is a renowned linear classification and visualization technique that produces projections that try to separate classes as much as possible. By coupling SC and LDA, analysts can gain insight regarding the discriminative power of the features by examining the length and orientation of the vectors that define a linear projection. In particular, analysts simply focus on the longest vectors that are oriented in the direction that better separates the classes, and choose one of these vectors according to their expertise. Thus, the visualizations allow clinicians to carry out feature selection interactively (see [21, 24–26]), where they can take advantage of their domain knowledge when selecting discriminative features.

## Methods

This section describes the dataset used in our study, and the visually guided approach for building classification trees.

### Dataset description

In this work we analyze data from healthy and chronic patients compiled during the year 2012 by the University Hospital of Fuenlabrada (UHF), in Madrid, Spain. The following information was recorded for each encounter of the patient with the public health system: age, gender, pharmacological dispensing (coded using the Anatomical, Therapeutic, Chemical (ATC) classification system [27]), and diagnoses collected by the hospital and by associated primary care centers. In particular, the diagnoses collected by the hospital were coded according to the International Classification of Diseases - 9^th^ revision-Clinical Modification (ICD9-CM) [28], whereas the diagnoses associated with primary care centers were coded in accordance with the International Classification of Primary Care (ICPC). Therefore, a clinical coding expert converted the ICPC codes into ICD9-CM codes, by considering free text written by primary-care physicians.

The ICD9-CM codes are categorized according to the disease type. Although the syntax of ICD9-CM codes allows us to consider categories and subcategories, in this work we omit subcategories in order to have a reasonable number of patients with the same codes. Thus, patients diagnosed in the same category (coded by three alphanumeric characters), but in different subcategories, will have the same diagnosis code. As a result, we consider a total of 1517 different ICD9-CM diagnosis codes. We followed the same approach for ATC codes. Although there are 3430 different ATC codes (composed of a combination of seven letters/digits), we have only worked with 746, by omitting the last two digits of the code, which refer to the chemical substance. All of these codes (i.e., features) are binary.

Based on demographic, diagnosis, and pharmacological dispensing data, each individual was assigned to a single mutually exclusive risk group (CRG). There are a total of 1080 categories of CRGs, identified by a five-digit number. The first digit refers to one of the following core health status groups: (1) healthy; (2) history of significant acute disease; (3) single minor chronic disease; (4) minor chronic diseases in multiple organ systems; (5) significant chronic disease; (6) significant chronic diseases in multiple organ systems; (7) dominant chronic disease in three or more organ systems; (8) dominant/metastatic malignancy; and (9) catastrophic. The second to fourth digits represent the base-CRG, whereas the fifth digit identifies the illness severity level.

In order to have a reasonable and sufficient number of patients in each category, in this paper we discarded the fifth digit (severity level), reducing the number of categories from 1080 to 242 (commonly known as base-CRG). In addition, out of these 242 base-CRG categories, we focused on only four related to EH and DM: CRG-5192 (hypertension), containing 12447 patients; CRG-5424 (diabetes), including 2166 patients; CRG-6144 (diabetes and hypertension), with 3179 individuals; and CRG-7071 (diabetes, hypertension, and other dominant chronic disease), which contains 547 patients. Furthermore, for comparison purposes, we also considered CRG-1000 (healthy), containing 46835 individuals.

**Fig. 1:**
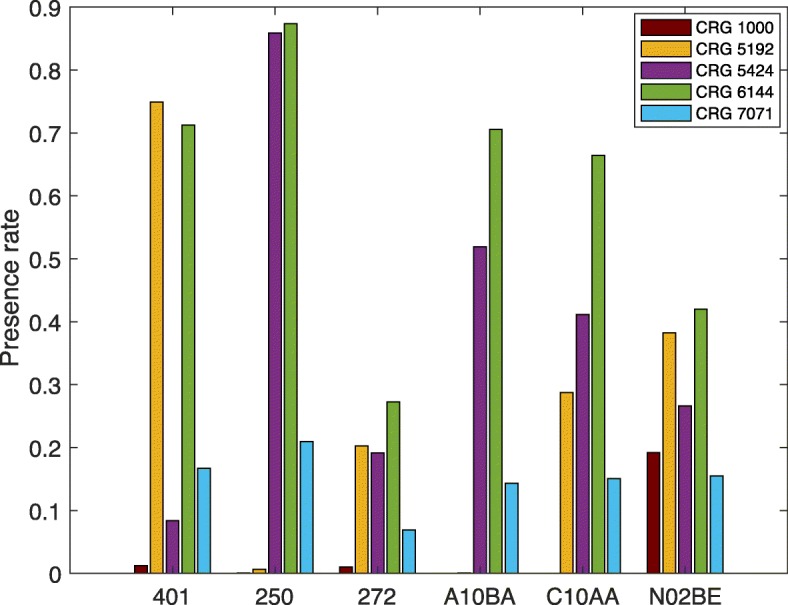
Presence rates. Presence rates for diagnoses codes ‘401’, ‘250’ and ‘272’ and ATC codes ‘A10BA’, ‘C10AA’ and ‘N02BE’. These features have the highest presence rates in the dataset

Figure 1 shows the presence rate per base-CRG for the three diagnoses and three ATC codes with the highest presence rates in the dataset. We define these rates as the ratio between the number of patients with a particular code in the base-CRG and the total number of patients in the same base-CRG. The most common diagnoses (ICD9-CM code) are EH (‘401’); DM (‘250’); and disorders of lipoid metabolism (‘272’). The most common drugs (ATC codes) are Biguanides (‘A10BA’), which is a blood glucose lowering drug; HMG CoA reductase inhibitors (‘C10AA’), used for the treatment of hypercholesterolemia; and Anilides (‘N02BE’), which includes drugs such as paracetamol. Regarding the colors used in the figures throughout this paper, we will use the following coding for the different classes: brown identifies CRG-1000; orange, CRG-5192; purple, CRG-5414; green, CRG-6144; and blue, CRG-7071.

As shown in Fig. 1, although CRG-1000 only contains healthy individuals, note that some of them have diagnoses and drugs related to DM and EH. In addition, around 25% of patients classified as hypertensive (CRG-5192) by the CRG system do not have the diagnosis code related to hypertension (‘401’). Furthermore, 11.8% of patients do not have ATC codes associated with hypertension, meaning that they are not undergoing any treatment. It is also interesting to note that about 15% of the patients in CRG-5424 do not have the diagnosis code related to DM (‘250’). This diversity in diagnoses and drugs for patients with the same chronic condition reflects the complexity of the task tackled in this paper.

### Visually guided classification trees

Statistical learning techniques are powerful tools for finding highly complex predictive functions that may be too difficult to model by a human being. Instead, these techniques “learn” the functions automatically by relying on examples provided in datasets. Among them, classification methods model the relationship between a finite set of samples, each characterized by a collection of *n* features, and a discrete target output category for each sample (its class or label). In this paper we will focus on the well-known classification trees, where the samples are patients and the output classes are provided by CRGs. Thus, we will build classifiers for predicting the health status (CRG) associated with a particular patient. Technically speaking, our classification trees learn predictive functions that map patients to CRG classes.

In this work we initially considered representing each patient by a set of *n* = 2265 features: gender (1), age (1), diagnosis codes (1517), and drug codes (746). However, we discarded those (around half) that had a zero count for all patients. In addition, in order to employ visualization methods effectively, we reduced the number of features even further by computing the entropy gain of each one according to Rauber and Steiger-Garção [29], and selecting the 50 features with the highest gain. Both diagnosis and drug codes were converted to binary features. Thus, they represent the presence or absence of a code, and not the number of times a patient has been diagnosed with a particular illness, or how many times a drug has been dispensed to a patient.

In general, when building a statistical learning classifier, the finite dataset of samples and labels is divided in two subsets: the training and test datasets. The training dataset is used to find the predictive function (i.e., to construct the model through a learning process), while the test set is used to evaluate the quality of the trained classifier. Generally speaking, a classifier is better the greater its accuracy predicting the classes of the samples in the test set. In other words, a good classifier should be able to generalize, providing correct outputs for samples that it has not seen in the training stage. However, in certain domains, such as in medicine, it is also important for analysts to interpret a classifier (i.e., understand how it works). For example, clinicians may need to comprehend and explain the decisions that the learning technique employs when classifying patients. In this regard, this paper focuses on classification trees, which are easier to interpret than the majority of statistical learning models.

Classification trees are methods used to partition a high-dimensional data space hierarchically, and are depicted graphically through a collection of nodes connected through branches in a hierarchical manner. All of the nodes are associated with some subset or region of the data space. Firstly, the root node corresponds to the entire data space. This initial space is then split into several disjoint regions that are related to the corresponding children nodes. This recursive structure is repeated at each node, partitioning the data space hierarchically. Note that a particular region of the data space related to a node will also be contained in the regions associated with its parent and its ascendant nodes.

**Fig. 2:**
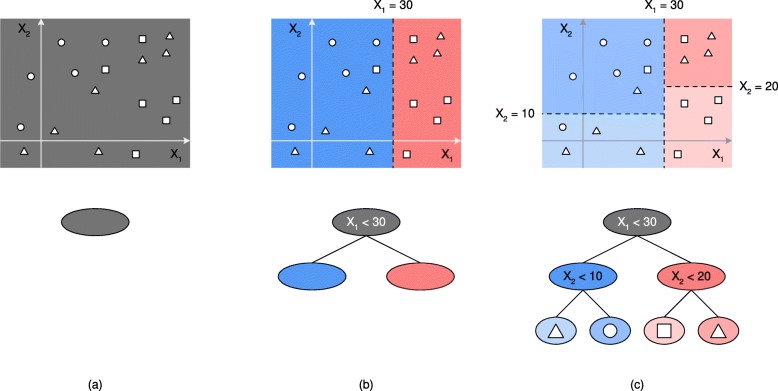
Data space partitioning by classification trees. The first step when creating a tree begins by considering the entire data space, represented in (**a**) for a two-dimensional dataset (with features *X*_1_ and *X*_2_), where at this point the tree only consists of a root node. The graphics also show samples belonging to three classes, which are coded as circles, squares and triangles. The first condition for splitting the data space is ‘ *X*_1_<30’, as shown in (**b**). In one region, all samples will have values for *X*_1_ less than 30, while in the other the values for *X*_1_ will be greater than or equal to 30. This creates two additional children nodes associated with each subregion, which can be further divided by considering mode conditions on the features. In (**c**), the two regions in (**b**) are split according to *X*_2_, generating two additional internal nodes and four leaves in the tree. Each leaf represents the most predominant class in their associated region

In order to partition the data space, the internal nodes of a classification tree (those that have children nodes) encode conditions on the features that specify how to partition the data space related to a node. Thus, the data spaces associated with each node will be split into two disjoint regions: those for which the condition is satisfied or not. The partitioning process halts at leaf (or terminal) nodes, which do not have any children nodes. Lastly, each leaf node specifies an output class, which is the one associated with the majority of the samples in the training set that belong to the region related to the leaf node. Figure 2 illustrates an example of the partitioning of a two-dimensional data space as a classification tree is constructed. Firstly, the entire data space (the plane created by features *X*_1_ and *X*_2_) is associated with the root node of the tree, and depicted as the gray area in (a). The samples belong to three classes, shown as circles, squares, and triangles. The first decision feature considered in the tree is *X*_1_, and the particular condition for splitting the data space is ‘ *X*_1_<30’, which splits the data space in two disjoint regions, as shown in (b). Subsequently, in (c) the two regions in (b) are split according to feature *X*_2_, which creates two additional internal nodes and four leaves in the tree, and generates a non-linear classification function. In this paper we will generate binary trees (the internal nodes will have two children nodes), where the condition will involve only one feature, and whose outcome will be either true or false.

Given a classification tree, the process of classifying a sample simply consists of applying the conditions at the nodes of the tree sequentially in a top-down manner, from the root node to a leaf. At each node, the decision of descending to a particular child node depends on the outcome of the node’s condition for the sample. Finally, the output of the classifier will be the class associated with the leaf node.

It is important to note that the size of a tree is related to the model’s complexity and its interpretability. In general, it is not convenient to provide neither big nor small trees, since they can generalize poorly. On the one hand, big trees can be too adapted to the samples used for learning, and can “memorize” training samples instead of generalizing for unobserved samples (this phenomenon is known as “overfitting”). Moreover, big trees can also be difficult to interpret. On the other hand, although small trees are quite interpretable, they are usually too simple and may not provide accurate predictions. According to Occam’s razor [30], simpler models are preferred over more complex ones whenever both types of models provide reasonable generalization (i.e., classification) capabilities.

**Fig. 3:**
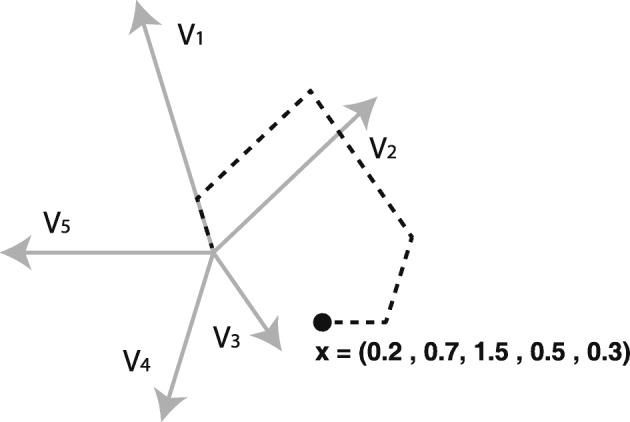
SC linear mapping. A data sample defined by five features **x** = (0.2, 0.7, 1.5, 0.5, 0.3) gets mapped onto the two-dimensional point **p** by adding scaled versions of the vectors **v**_*i*_, where the scaling factors are the values of **x** associated with each feature

In this paper we propose to construct classification trees in an interactive and visually guided way, where clinicians can benefit from their domain knowledge. Instead of automatically obtaining the feature to better classify samples in a node (as is carried out by the majority of the methods that generate classification trees), in our case clinicians can select the most suitable feature by observing a SC plot that represents the different classes in the data. SC is a multidimensional visualization method that generates linear mappings from a high *n*-dimensional data space onto a lower two-dimensional plane in order to represent the data graphically. In particular, it constructs plots through a set of two-dimensional vectors **v**_*i*_, for *i*=1,…,*n*, with a common origin point, where **v**_*i*_ is associated with the *i*-th feature (see Fig. 3). The interpretation of the axis vectors is straightforward: the orientation determines the direction in which a feature increases, and the length specifies the amount of contribution of a particular feature in the resulting plot.

SC allows analysts to generate arbitrary layouts by interactively modifying the axes. This can also be used to couple SC with any linear projection method such as LDA [21, 22]. LDA is a popular linear statistical method used to find a combination of features that separates the samples belonging to different classes, by maximizing the ratio between the inter-class and intra-class variance.

In this paper, we combine SC with LDA in order to provide a projection that separates the samples optimally according to their classes. In addition, the visualizations allow analysts to determine the contribution of the different features to the classification, by examining the lengths and orientations of the SC axis vectors. Although the longer axis vectors are more relevant to the plot in general [21], their orientations should also be considered when determining the most discriminative features that contribute more to separating the classes [26].

Clinicians can therefore examine the two-dimensional vectors associated with the variables when deciding which is the most important one for separating certain classes, according to the plot and their expert knowledge. Once the variable is selected, a new internal tree node is created and samples in that node are assigned to different classes. When a feature is binary (as most in our dataset), the division is simply carried out according to the two values of the feature. In the case of non-binary characteristics (i.e. age), the cut-off value is provided by the highest information gain ratio. In order to construct the classification tree, the clinicians recursively analyze each subset of data obtained through the splitting process, until they consider that most of the samples in a resulting subset belong to the same class, or when it contains just a few samples. In these cases a leaf node is created.

For example, we analyzed which features contribute more to creating a simple classification tree among patients with CRGs-5192-6144-7071. Regarding the training set to build the tree, we randomly chose 80% of the available dataset. However, since statistical learning techniques are affected by an imbalance in the number of samples per category, we followed an undersampling strategy to balance them, by selecting the same number of samples from the minority class. The size of the training set was therefore fixed by the number of samples of the class with less instances (CRG-7071), multiplied by the number of classes.

**Fig. 4:**
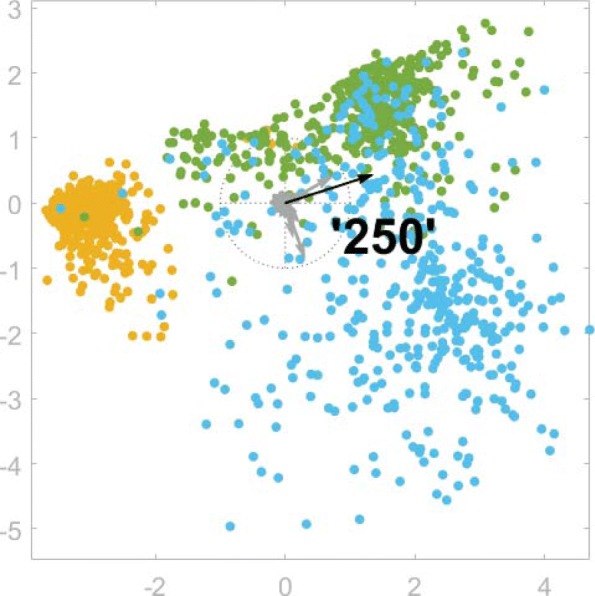
Combination of SC and LDA for CRGs 5192-6144-7071. The mapped points (dots) correspond to an LDA plot, which separates the classes in an optimal way. In addition, the SC radial axis vectors (the specific set of vectors is chosen in order to generate the LDA mapping when applying SC) provide information about the features. Their length and orientation indicate the contribution of the features to the LDA class separation task. In this case the longest vector corresponds to the diagnosis code ‘250’, and points to the direction that better separates the points in CRG-5192 (orange) from the rest. The plot also shows that points of the CRG-6144 (green) and CRG-7071 (blue) classes are highly overlapped

Figure 4 shows an example of the SC plot associated with LDA for the training data of CRGs 5192-6144-7071, where the classes are separated fairly well (note that overlaps occur frequently when working with real-world datasets). In this plot clinicians can identify the different health statuses graphically, and can select the most important and discriminative features when creating the classification tree. In this case, ‘250’ (DM) is the longest axis vector, and is oriented in the direction separating CRG-5192 from the rest. The ICD9-CM code ‘250’ is therefore the most suitable feature for dividing the dataset. This is in accordance with domain knowledge, since the diagnosis code ‘250’ makes it possible to distinguish between patients belonging to CRG-6144-7071 (two or more comorbidities, including DM) and CRG-5192 (hypertensive patients with absence of DM).

**Fig. 5:**
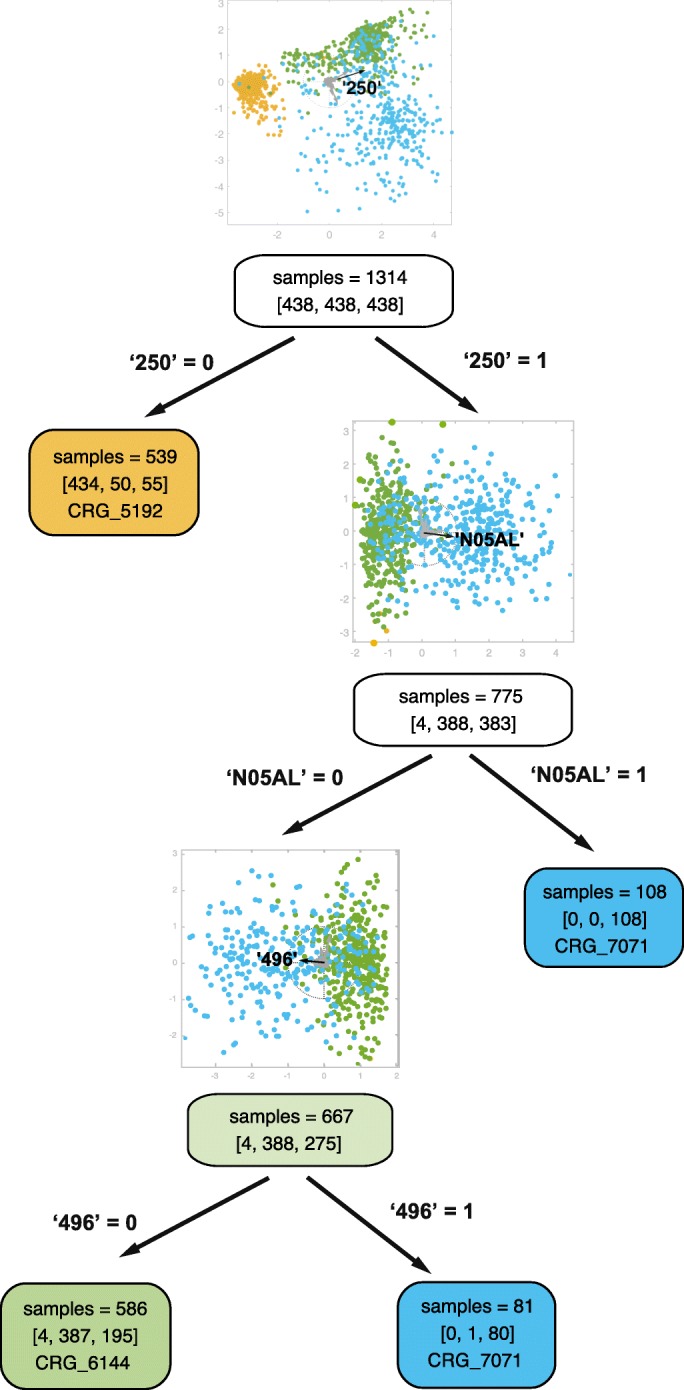
Visually guided classification tree. Example of the design of a classification tree guided by visual LDA plots. In each node we show the total number of samples contained in its associated region, and in square brackets the number of samples per class [CRG-5192, CRG-6144, CRG-7071]. Initially the dataset is balanced (there are the same number of samples per CRG). The root node splits the dataset in two by the presence or absence of the diagnosis code ‘250’ in a guided way, considering the LDA plot above the node (also shown in Fig. 4). Most of the samples of the left branch belong to CRG-5192, and thus this node can be a leaf node representing that class if the clinician considers it appropriate. In the right branch, a new LDA plot is computed with the data subset for which the code ‘250’ =1 (i.e., is present). In this case, the drug code ‘N05AL’ is the feature that better separates samples from CRG-6144 and CRG-7071. Thus, we consider a new split based on this feature. Its right branch (presence) only contains CRG-7071 samples, and therefore constitutes a leaf node. Subsequently, we generate a new LDA plot with the remaining data for which ‘N05AL’ is absent. In this case, the diagnosis code ‘496’ is the longest axis vector that points towards the direction that better helps to separate the classes, and it is selected to split the corresponding region. Since most of the samples in the right branch belong to CRG-7071, we consider that it should be a leaf node. In the left branch, this process could be performed recursively until each node is defined as a leaf. In this case, since most of the samples belong to CRG-6144, the process could be halted, creating the leaf node

As illustrated in Fig. 5, the dataset used in Fig. 4 is split in two subsets by the presence or absence of the ICD9-CM code ‘250’. Below the LDA plot we show the corresponding decision node, which we have colored according to the predominant class. The hue is determined by the specific class, and its lightness depends on the percentage of patients belonging to the class (the higher the percentage, the darker the color). Finally, we use white to color nodes when there is not a clearly predominant class. Since most of the patients without the diagnosis code ‘250’ belong to the class CRG-5192, this subset could be considered as a leaf node. Instead, since there is not a clear majority class among patients who have the code ‘250’, a new LDA plot can be displayed in order to select another discriminative feature, which will cause the tree to grow in that branch. In this case we observe a similar number of samples belonging to CRG-6144 and CRG-7071. We can see that the drug ‘N05AL’ (antipsychotics) is the longest axis vector in the direction separating both classes. All patients in the training dataset who have codes ‘250’ and ‘N05AL’ belong to CRG-7071, leading again to a leaf node. For the samples where the drug code ‘N05AL’ is absent, we can display a new LDA plot. In that case the diagnosis code ‘496’ (chronic airway obstruction) is the most suitable feature to separate the classes. For this new subset of samples, the ICD9-CM code ‘496’ indicates that most of them belong to CRG-7071. Observe that in each tree branch the LDA visualization process can be carried out recursively, allowing analysts to select the axis vector (i.e., the feature) to be used for splitting the subsets related to the nodes of the tree. In this simple example we halted the process at this point, creating the last leaf node.

## Results

In this section we present the results provided by the visually guided classification trees. We analyzed performance in terms of accuracy, F1-score and confusion matrices, and drew clinical conclusions regarding the features considered in each decision tree. Specifically, clinicians constructed five visually guided decision trees to discriminate patients among health statuses: (1) CRGs 5192-6144-7071, in other words, classify patients into CRG-5192, CRG-6144, or CRG-7071; (2) CRGs 1000-5192-6144-7071; (3) CRGs 5424-6144-7071; (4) CRGs 1000-5424-6144-7071; and (5) CRGs 1000-5192-5424-6144-70717.

For simplicity, instead of showing all of the LDA plots needed to create the trees, we only show the features selected by the clinicians in the decision nodes. Each decision node generates two branches when evaluating a particular feature: the left branch refers to samples that satisfy the decision rule displayed within the internal node, whereas the right branch represents the subset of samples that do not satisfy the node’s condition. Leaf nodes show the name of the predominant base-CRG class, and the percentage of the node samples belonging to it (in brackets). In addition, for a better visual understanding of the tree, we colored every node according to its predominant associated class, setting its lightness in accordance with the percentage of samples labeled with the majority class.

### Evolution of hypertension

**Fig. 6:**
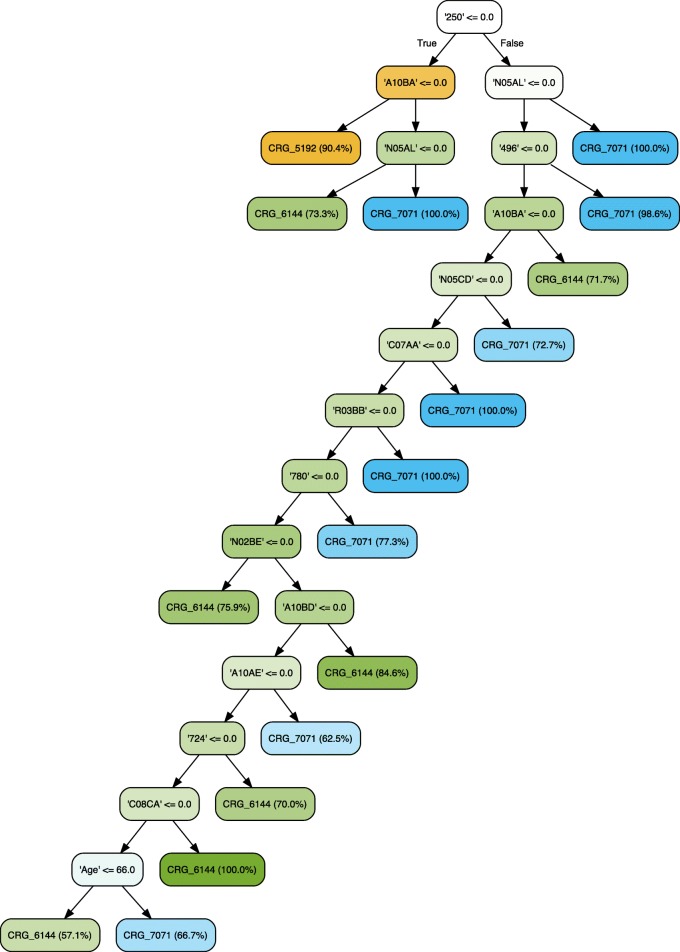
Classification tree considering age, gender, and diagnosis and drug codes for CRGs 5192-6144-7071. The tree shown in this figure is more complex than the one shown in Fig. 5, which mainly identifies patients in CRG-5192 in the left branch of the root node. The extension of this branch with the ATC code ‘A10BA’ allows a more detailed classification, allowing us to identify patients who also suffer from DM. The right branch of the root node tries to establish the conditions for separating patients from CRG-6144 and CRG-7071, where the diagnosis code ‘496’ and/or ATC code ‘N05AL’ are the most relevant features for discriminating between these two CRGs

We firstly explored the potential to predict the evolution of hypertension. Towards that goal, clinicians built a decision tree supported by visualizations when only considering CRGs 5192-6144-7071 (see Fig. 6). Observe that this tree is an extension of the example shown in Fig. 5. Similarly to Fig. 4, the ICD9-CM code ‘250’ is the most discriminative feature since it allows us to separate the CRG-5192 class from the rest. In contrast, the diagnosis code ‘401’ does not appear since it is generally present in the three considered classes.

**Fig. 7:**
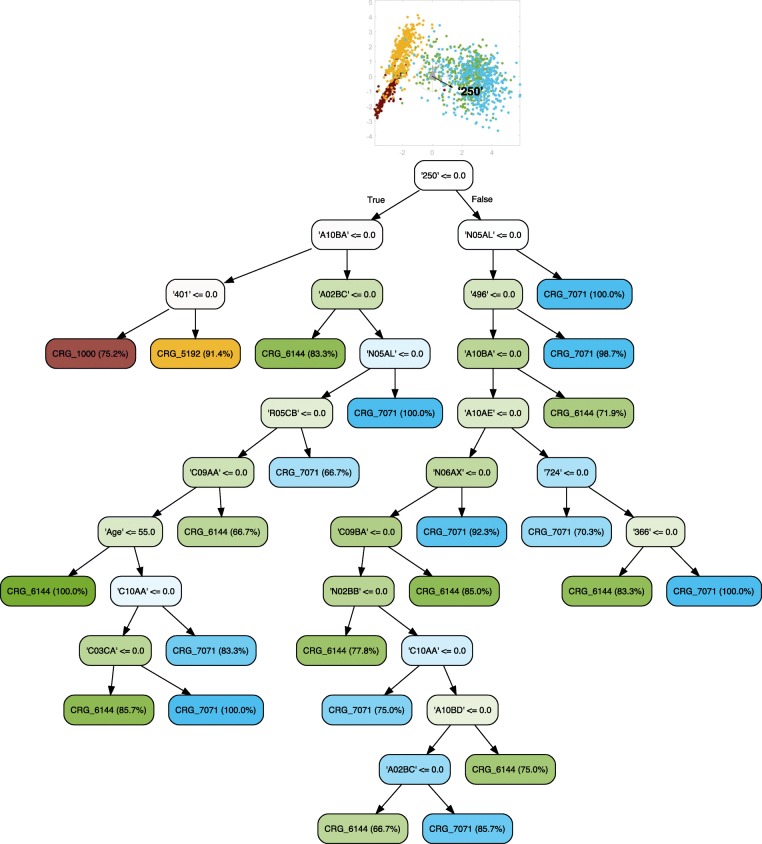
Classification tree considering age, gender, and diagnosis and drug codes for CRGs 1000-5192-6144-7071. The LDA plot at the top of this figure is shown to understand why clinicians chose the diagnosis code ‘250’ as the condition in the root node. The plot shows that samples of CRGs 1000-5192 can be easily separated from those of CRGs 6144-7071 by means of the longest axis vector, representing the diagnosis code ‘250’ (DM). This tree is similar to the one constructed for CRGs 5192-6144-7071 (see Figure 6). The main difference is found in the left branch of the root node (non diabetic patients), which now includes healthy patients (CRG-1000). The most discriminative feature between CRG-1000 and CRG-5192 is the diagnosis code ‘401’ (hypertension), which is associated with that chronic condition

The tree obtained when including healthy patients (CRGs 1000-5192-6144-7071) is shown in Fig. 7. The initial LDA plot at the top of the tree provides insight regarding which variables are most discriminative (i.e., more relevant regarding class separation). In the LDA plot there are two clearly separated groups of elements on the left that represent CRG-1000 and CRG-5192. CRG-7071 is located on the right, although it is clearly overlapped with CRG-6144. In this scenario, the vector for diagnosis code ‘250’ is the longest one separating the first two classes from the rest, and this is the reason why clinicians selected this variable to start the tree. Afterwards, they continued the process shown below to create the classification tree according to the LDA plots. In contrast to Fig. 6, the diagnosis code ‘401’ allows clinicians to separate the healthy patients from the chronic hypertensive ones, and for this reason it appears in Fig. 7.

### Evolution of DM

**Fig. 8:**
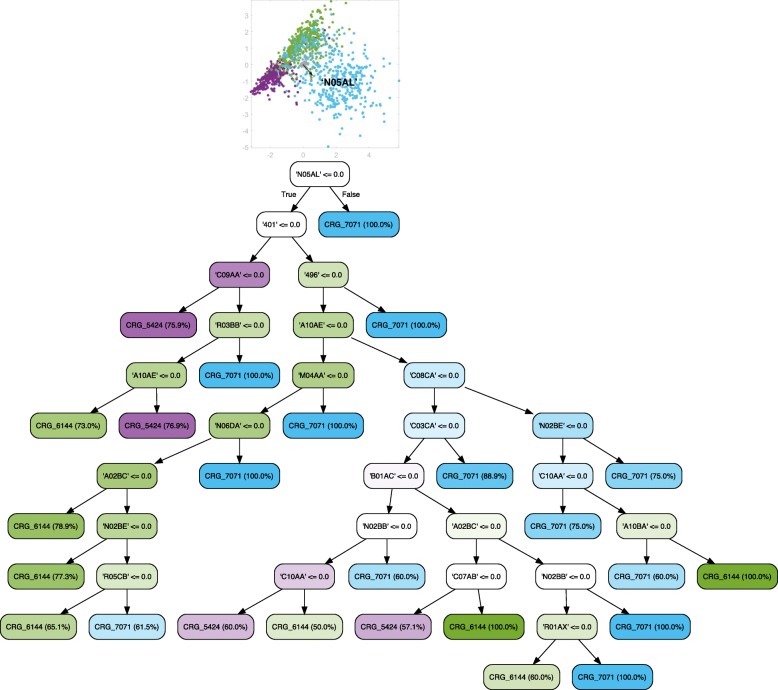
Classification tree considering age, gender, and diagnosis and drug codes for CRGs 5424-6144-7071. The initial LDA plot shows that samples of the different CRGs appear overlapped. It may be because corresponding patients share a common chronic condition (diabetes). The absence of diagnosis code ‘401’ (hypertension) and drug code ‘C09AA’ (based on the treatment of mild-moderate hypertension) leads directly to CRG-5424, since it is the only CRG that does not consider the chronic condition EH. Furthermore, the ATC code ‘N05AL’ is the most discriminative feature for identifying CRG-7071 patients, which is the class that corresponds to patients with the highest number of chronic conditions (among the considered CRGs)

**Fig. 9:**
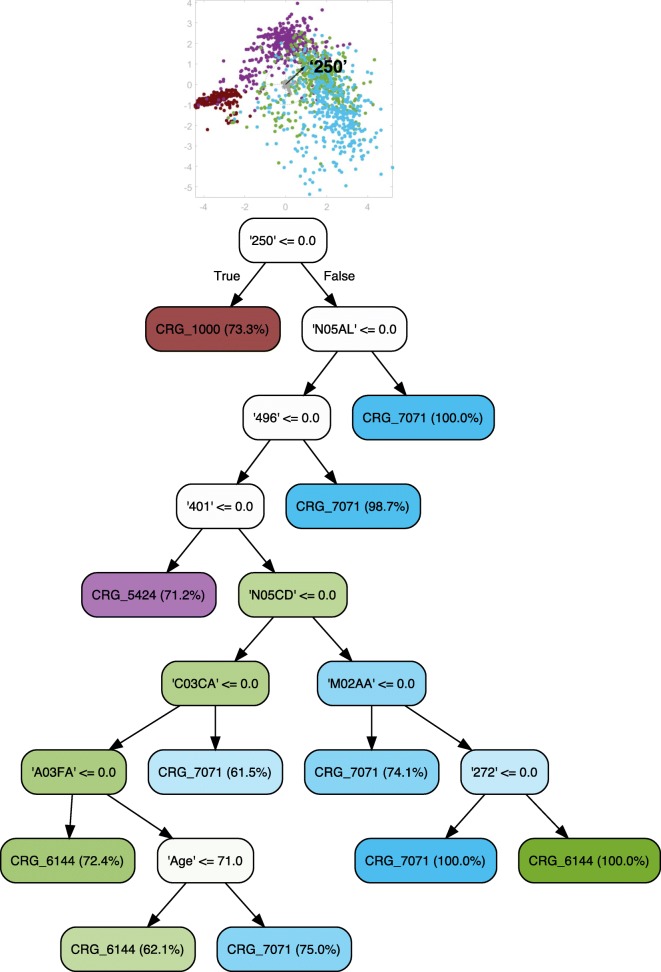
Classification tree considering age, gender, and diagnosis and drug codes for CRGs 1000-5424-6144-7071. Clinicians can easily separate patients belonging to CRG-1000 from the rest based on the diagnostic code ‘250’, since its absence identifies healthy individuals. However, its presence in conjunction with the absence of codes ‘401’, ‘496’ and ‘N05AL’ leads to patients in CRG-5424. The presence of the last three codes identifies patients in CRGs 6144 and 7071

We secondly explored the potential to predict the evolution of DM considering individuals categorized by the CRG system as diabetic, and where in some cases they may have multiple chronic conditions (see Fig. 8). The classification model was extended by constructing a tree that also considered the group of healthy patients (see Fig. 9 for details). Both LDA plots show a clearly defined group for CRG-5424 on the left. When CRG-1000 is included, it is the left-most group. In addition, patients belonging to CRG-6144 and CRG-7071 are still overlapping, showing that they share similar values for many features. Regarding the diagnosis code ‘250’, on the one hand, it does not appear in Fig. 8 since all of the patients analyzed are diabetic, and therefore the code ‘250’ is not suitable for separating the classes. Instead, the use of antipsychotics (‘N05AL’) was the most important feature for clinicians because it is the longest vector in the direction that separates most of the patients of CRG-7071 from the rest. On the other hand, similarly to the trees associated with hypertensive patients, the diagnosis code ‘250’ is the most discriminative feature when including CRG-1000, as shown in Fig. 9. In this case, it is the most relevant vector for separating healthy patients from the rest.

### Evolution of DM and EH

**Fig. 10:**
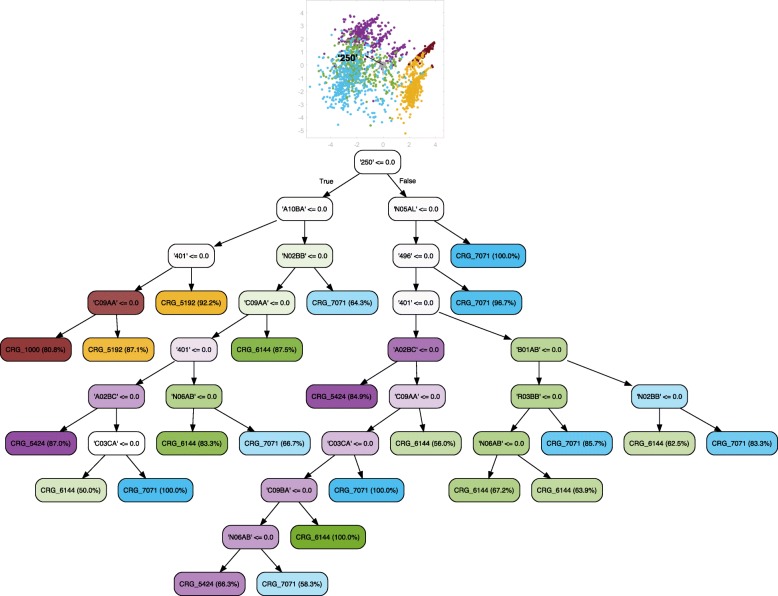
Classification tree considering age, gender, and diagnosis and drug codes for CRGs 1000-5192-5424-6144-7071. The LDA plot shows that samples in CRGs 1000 and 5192 are separated from the rest (see the right part of the plot). Note that these CRGs are associated with the leaf nodes on the left branch of the tree. As expected, the absence of diagnosis codes related to DM (‘250’) and EH (‘401’), together with the absence of drug codes related to DM (‘A10BA’) and EH (‘C09AA’) leads to healthy patients, which is consistent with domain knowledge. The presence of the diagnosis code ‘401’ or the presence of the drug code ‘C09AA’ identifies patients in CRG-5192. Also, in accordance with previously designed trees, the presence of diagnosis code ‘496’ or drug code ‘N05AL’ helps us to identify the patients belonging to CRG-7071. Since this is the tree that considers more health statuses, the relationships among features and CRGs may be more difficult to interpret, and to verify whether they agree with current clinical knowledge

Since EH is related to DM, we also built a tree encompassing healthy individuals and patients suffering from EH and DM. As shown in Fig. 10, the LDA plot is more complex than the previous ones. Nevertheless, there are some small groups on the right, like CRG-1000 and CRG-5192. These can be easily identified as the groups for which code ‘250’ is absent, since the corresponding axis vector is the longest that points towards the left (note that the direction in which the vector points indicates presence). Thus, the diabetes code ‘250’ was selected to appear at the first level of the tree. At the second level we have drug codes that allow clinicians to classify the patients better. On the one hand, the absence of code ‘250’ is complemented by the presence of the drug code ‘A10BA’ (drug for lowering blood glucose), which allows us to identify patients suffering from diabetes. Instead, the absence of both of these codes allows us to identify non-diabetic patients (CRG-1000 and CRG-5192). Note that in this case, the presence of EH (‘401’) is associated to hypertensive patients (CRG-5192). On the other hand, the presence of codes ‘250’ and antipsychotics (‘N05AL’) helps us to identify the patients in the CRG-7071 class. As expected, the diagnosis code of chronic airway obstruction (‘496’) leads to CRG-7071.

### Classification performance

We analyzed the performance of the visually guided trees from a predictive perspective in terms of accuracy on a test set [31]. This set was obtained by considering the same number of samples by class, which was 20% of the size of the minority class (CRG-7071). Since the dataset is unbalanced, and in order to avoid biases in performance, we randomly selected 50 different test sets. Thus, we report mean (and standard deviation) accuracy scores over 50 trials.

Table 1 shows the performance of the trees for the five mentioned scenarios. As expected, we obtained better results when considering healthy patients since they have a clearly different pattern of features from that of patients suffering from chronic conditions. Thus, it is easier to detect healthy patients, which improves the overall classification accuracy. The mean accuracy for discriminating CRGs related to hypertensive patients when considering healthy patients (CRG-1000) was 87.45%, while it was 82.19% when not considering them. These performance measurements are in line with those obtained with a traditional classification tree over the same dataset. For instance, the work in [13] considers the automatic algorithm C4.5, which chooses the features providing the highest information gain ratio when creating the decision nodes. This algorithm is commonly used to build classification trees in a non-guided way. Instead of using the C4.5 algorithm, in our approach clinicians rely on visualizations and can take advantage of their domain knowledge when selecting features in the decision nodes.

**Table 1:** Mean and standard deviation (in brackets) for the classification accuracy on 50 trials when using the trees to discriminate among different classes (columns)

Features	CRGs 5192-6144-7071	CRGs 1000-5192-6144-7071	CRGs 5424-6144-7071	CRGs 1000-5424-6144-7071	CRGs 1000 5192-5424-6144-7071
Age, gender, ICD9 and ATC codes	82.19 (1.92)	87.45 (1.66)	76.04 (1.95)	85.32 (2.05)	78.93 (1.59)

We also analyzed the performance of the classification tree through confusion matrices, which are *C*×*C* tables that show true versus predicted classes, for classification problems involving *C* different classes. Entries in the diagonal cells report the percentage of samples with correct predictions (accuracies), whereas those on the off-diagonal cells represent percentages of misclassified samples. We also present the corresponding F1 score (in particular, means and standard deviations over 50 trials) as another measure of the accuracy of each model. This measure takes into account both the precision (true positive results obtained divided by the total predicted positive results) and the recall (true positive results divided by all of the elements that should have been identified as positive). The F1 score is a value between 0 and 1, where 1 indicates perfect classification performance.

Table 2 shows the confusion matrix for the trees constructed to classify samples in CRGs 5192-6144-7071, both for the extended (final stage in Fig. 6) and for the nonextended trees (first stage in Fig. 5). In this table the rows are the true classes (provided by the CRGs), whereas the columns are the classes predicted by our classifier. As expected, we obtained worse results with the tree created in the first stage since it could be too small to capture the complexity of the dataset, specially for the class with more chronic conditions (GRG-7071). In contrast, the most complex tree (final stage) correctly classified 99.54% of the patients in CRG-5192 (on average), misclassifying only 0.40% in CRG-6144, and 0.05% in CRG-7071. For CRG-6144 the percentage of patients correctly classified is 90.37%, where 3.39% and 6.24% of the patients were predicted to belong to CRGs 5192 and 6144, respectively. We obtained lower accuracies when considering the more complex scenario involving more comorbidities. In that case, only 61.86% of the patients in CRG-7071 were correctly classified, where almost 40% of them were associated to CRG-6144, and around 8% belonged to CRG-7071.

**Table 2:** Confusion matrix for CRGs 5192-6144-7071, in the first and final stages of the classification tree, showing mean (and standard deviation) on 50 trials

CRG		Predicted		
		5192	6144	7071
5192	First stage	**99.82 (0.80)**	0.18 (0.68)	0 (0)
	Final stage	**99.54 (0.76)**	0.40 (0.74)	0.05 (0.21)
6144	First stage	12.86 (3.06)	**86.99 (3.12)**	0.15 (3.86)
	Final stage	3.39 (1.66)	**90.37 (2.82)**	6.24 (2.38)
7071	First stage	14.7 (3.33)	47.42 (5.02)	**46.88 (4.49)**
	Final stage	7.46 (1.78)	39.68 (5.03)	**61.86 (4.99)**

F1 score for the first stage: 0.76 (0.018). F1 score for the final stage: 0.85 (0.016)

Comparing Tables 2 and 3 we observe that predicting hypertension is an even more challenging task if healthy patients are considered. Indeed, only 58.55% of the patients encompassed in CRG-7071 were predicted correctly (see Table 3). The predictions were also lower for CRGs 5192 and 6144, with percentage classification accuracies of 73.65% and 89.85%, respectively. Lastly, observe that healthy patients are classified exceptionally well.

**Table 3:** Confusion matrix for the CRGs 1000-5192-6144-7071 classification tree, showing mean (and standard deviation) on 50 trials

CRG	Predicted			
	1000	5192	6144	7071
1000	**99.82 (0.80)**	1.14 (1.12)	0.04 (0.17)	0.02 (0.13)
5192	25.03 (4.17)	**73.65 (4.11)**	0.84 (0.86)	0.02 (0.13)
6144	2 (1.05)	0.73 (0.78)	**89.95 (2.98)**	7.49 (2.27)
7071	3.78 (1.38)	2.86 (1.52)	34.81 (3.91)	**58.55 (4.00)**

F1 score: 0.80 (0.016)

Similar results are obtained when considering DM (see Tables 4 and 5 for details). Finally, Table 6 shows the confusion matrix related to the classification tree for all analyzed CRGs 1000-5192-5424-6144-7071. The classification accuracy is higher for healthy, hypertensive, and diabetic patients than for those in CRG-7071 and CRG-6144. These results are in accordance with the corresponding LDA plot, where the CRGs-7071-6144 are highly overlapping.

**Table 4:** Confusion matrix for the CRGs 5424-6144-7071 classification tree, showing mean (and standard deviation) on 50 trials

CRG	Predicted		
	5424	6144	7071
5424	**91.19 (2.83)**	8.09 (2.67)	0.72 (0.67)
6144	17.95 (3.32)	**74.72 (3.51)**	7.34 (2.39)
7071	17.67 (3.15)	21.78 (3.66)	**60.55 (4.66)**

F1 score: 0.755 (0.02)

**Table 5:** Confusion matrix for the CRGs 1000-5424-6144-7071 classification tree, showing mean (and standard deviation) on 50 trials

CRG	Predicted			
	1000	5424	6144	7071
1000	**99.96 (0.17)**	0.02 (0.13)	0.02 (0.14)	0 (0)
5424	3.30 (1.66)	**77.41 (4.11)**	18.39 (3.45)	0.90 (0.74)
6144	2.88 (1.65)	11.68 (10.72)	**78.68 (3.90)**	7.72 (2.72)
7071	6.84 (2.51)	9.01 (2.37)	27.36 (4.05)	**56.79 (4.20)**

F1 score: 0.78 (0.017)

**Table 6:** Confusion matrix for the CRGs 1000-5192-5424-6144-7071 classification tree, showing mean (and standard deviation) on 50 trials

CRG	Predicted				
	1000	5192	5424	6144	7071
1000	**98.70 (1.01)**	1.23 (1.04)	0.07 (0.25)	0 (0)	0 (0)
5192	18.5 (4.37)	**82.26 (4.05)**	0.24 (0.44)	0.50 (0.79)	0.04 (0.17)
5424	2.92 (1.33)	0.37 (0.61)	**83.49 (3.33)**	10.94 (3.14)	2.29 (1.50)
6144	1.32 (1.00)	1.52 (1.08)	16.00 (3.36)	**70.18 (4.09)**	10.97 (2.95)
7071	2.57 (1.39)	3.80 (1.49)	6.31 (1.94)	27.19 (2.75)	**60.13 (3.46)**

F1 score: 0.79 (0.016)

### Clinical insights

Finally, we analyzed in detail the set of selected features and their position in a tree when classifying patients according to a health status. Table 7 shows the selected features in the tree nodes (first column), their description when they correspond to a code (second column), and their presence in the trees (the next five columns, where each one corresponds to a different tree). For each tree, blank cells indicate that the feature has not been selected in that tree, while the numbers in brackets denote the tree levels where the feature is considered.

**Table 7:** List of features appearing in the constructed trees for discriminating among different classes (the five columns on the right)

Feature	Description	5192-6144-7071	1000-5192-6144-7071	5424-6144-7071	1000-5424-6144-7071	1000-5192-5424-6144-7071
250	Diabetes mellitus	(1)	(1)		(1)	(1)
272	Disorders of lipoid metabolism				(7)	
366	Cataract		(7)			
401	Essential hypertension		(3)	(2)	(4)	(3,4,5)
496	Chronic obstructive pulmonary disease	(3)	(3)	(3)	(3)	(3)
724	Other and unspecified disorders of back	(12)	(6)			
780	General symptoms	(8)				
A02BC	Proton pump inhibitors		(3,11)	(7,8)		(5,6)
A03FA	Propulsives				(7)	
A10AE	Insulins and analogues for injection, long-acting	(11)	(5)	(4,5)		
A10BA	Blood glucose lowering drugs, excl. Insulins	(2,4)	(2,4)	(8)		(2)
A10BD	Combinations of oral blood glucose lowering drugs	(10)	(10)			
B01AB	Heparin group. Antithrombotic agents					(5)
B01AC	Platelet aggregation inhibitors excl. heparin			(7)		
C03CA	Sulfonamides, plain. High-ceiling diuretics		(9)	(6)	(6)	(7)
C07AA	Beta blocking agents, non-selective	(6)				
C07AB	Beta blocking agents, selective			(9)		
C08CA	Selective calcium channel blockers with mainly vascular effects. Dihydropyridine derivatives	(13)		(5)		
C09AA	ACE inhibitors, plain		(6)	(3)		(4,6)
C09BA	ACE inhibitors and diuretics		(7)			(8)
C10AA	HMG coa reductase inhibitors		(8,9)	(7,9)		
M02AA	Antiinflammatory preparations, non-steroids for topical use				(6)	
M04AA	Preparations inhibiting uric acid production			(5)		
N02BB	Other analgesics and antipyretics. Pyrazolones		(8)	(8,9)		(3,6)
N02BE	Other analgesics and antipyretics. Anilides	(9)		(6,8)		(6)
N05AL	Antipsychotics. Benzamides	(2,3)	(2,4)	(1)	(2)	(2)
N05CD	Hypnotics and sedatives	(5)			(5)	
N06AB	Selective serotonin reuptake inhibitors					(6,7)
N06AX	Antidepressants		(6)			
N06DA	Antidepressants			(6)		
R01AX	Other nasal preparations			(10)		
R03BB	Anticholinergics	(7)		(4)		(6)
R05CB	Mucolytics		(5)	(9)		
Age		(14)	(7)		(8)	

The numbers in parenthesis correspond to the level where the feature appears in the tree (where the root node is at level 1)

The level denotes the depth of a node in the tree, where the root node is at level 1, its children are at level 2, and so on. We can interpret that the lower the level of a node, the more discriminative its related feature is regarding the entire data set. Alternatively, since nodes in higher levels are associated with fewer samples, their corresponding decision feature is likely to be less discriminative. Lastly, features with more than one number in parenthesis appear at several different levels of the tree.

Note that the presence of the diagnosis code ‘250’ (DM) always appears in the first node, when discriminating healthy patients versus chronic patients who suffer from one or both conditions (hypertension/diabetes), with the exception of CRGs 5424-6144-7071. The reason is that patients in these three classes are diabetic, and the code ‘250’ is not relevant for discriminating among these groups. A similar situation occurs regarding the presence of code ‘401’ when only CRGs 5192-6144-7071 are considered.

It is interesting to point out that the diagnosis code ‘496’ (chronic obstructive pulmonary disease) is quite discriminative in all trees. Since CRG-7071 includes patients suffering from EH, DM, and other dominant chronic diseases, it is likely that code ‘496’ appears for characterizing the other dominant chronic diseases.

Table 7 also shows the drug codes appearing in the trees. Note that the number of drug codes is much higher than the number of diagnosis codes, though some of the drug codes are used to treat the same disease. Thus, as an example, ATC codes ‘A10AE’, ‘A10BA’ and ‘A10BD’ are used for treating DM, while ATC codes ‘C03CA’, ‘C07AA’, ‘C07AB’, ‘C09AA’, ‘C09BA’ are employed for EH. The ATC codes ‘R01AX’, ‘R03BB’ and ‘R05CB’ are drugs related to chronic obstructive pulmonary disease, and allow us to discriminate patients associated with CRG-7071. Furthermore, it is also interesting to point out that the ATC codes related to antipsychotics and antidepressants (such as ‘N05AL’, ‘N05CD’, ‘N06AB’, ‘N06AX’ and ‘N06DA’) allow us to identify patients in CRG-7071. Note that mental disorders can be considered to be another dominant chronic disease.

The use of the therapeutic group denoted by ATC codes ‘B01AB’ and ‘B01AC’, which comprises antithrombotic agents and heparins, is indicated in situations of high risk of blood clots, leading to severe pathologies related to the cardiovascular and cerebral system. These drugs are prescribed to patients with serious chronic conditions, being the alteration of the cardiac rhythm (atrial fibrillation) the most frequent one. The presence of any of these drugs may help clinicians to discriminate between CRG-6144 and CRG-7071.

Finally, we analyzed the association between age and health statuses. The mean (and standard deviation) age of patients is the following: 26 (15) for CRG-1000, 54 (14) for CRG-5192, 49 (15) for CRG-5424, 62 (11) for CRG-6144, and 66 (12) for CRG-7071. As expected, healthy patients are younger than those with chronic conditions, and the number of comorbidities increases with the age of the patients. Analyzing the constructed trees, we can see that, in general, the feature ‘age’ is very close to a leaf node when separating between CRG-6144 and CRG-7071 (the older the patients, the worse their health status). Regarding gender, the percentage of men and women is similar in our population both per each CRG and among CRGs. This may be the reason why we did not find the gender feature in the constructed trees, which suggests that it is not a relevant feature for discriminating among health statuses.

## Discussion

The term “chronic disease” or “chronic condition” is used to identify a health status that is persistent or has a long duration and slow progression. The most prominent chronic diseases are DM, EH, chronic obstructive pulmonary disease, and cardiovascular diseases, among others. There are key risk factors for these chronic conditions, such as overweight, physical inactivity, or tobacco and alcohol use, among others. The availability of data related to these factors is a challenge since they are rarely registered in the EHR. However, other information in the EHR such as diagnoses and drugs are compiled in a more reliable way and are also registered according to international codes. These codes can be used in the patient classification systems (PCSs) to identify both the health statuses as well as patients with high consumption of health resources. One of the most common PCSs are CRGs, which are oriented towards the identification of chronic patients. In this work, we have characterized patients suffering from chronic conditions such as DM and EH by using diagnosis and drug codes, together with age, gender, and the health status provided by the CRG system.

Statistical learning methods provide a way to create models (linear or nonlinear) to find patterns in data, and have shown potential to extract new knowledge from clinical datasets [32, 33]. In the health care domain, the use of linear regression has been commonly used since it provides insight about the relevance of the features [34]. However, researchers in this domain are currently shifting towards inherently nonlinear techniques in order to find higher-order interactions or nonlinear relationships. In general, they enhance the linear approaches, but they are barely interpretable. However, classification trees can provide nonlinear models that are easy to interpret, and they have been used effectively in different application domains [35, 36], including health care [13]. There are different automatic methods for constructing classification trees [14, 37, 38]. The main idea is to recursively choose the feature that provides the highest information gain ratio, gini impurity, or variance reduction, to split the branches. Although the construction of the tree using these criteria is automatic, they do not allow analysts to take advantage of their expertise. Instead, the main advantage of our proposal is that the construction of the classification trees is supported by statistical visual representations of the data, where analysts choose features in a visually guided manner. Thus, clinicians can select the most suitable feature to be used in the trees by taking into account their domain knowledge and the relevance of each feature from a clinical viewpoint. In our approach, the criterion of information gain or gini ratio are not considered to select the splitting feature. This could be a limitation, since different clinicians may select different features, and therefore, different trees could be built.

The trees constructed following our methodology provide accuracy values similar to those obtained with automatic methods when using the same dataset [13]. Accuracies are higher as patients have fewer chronic conditions (CRG-5192 and CRG-5424 encompass patients with only one chronic condition). As expected, the ICD9-CM code ‘250’ (DM) is present in most of the patients in CRG-5424. The same applies for hypertensive patients with the ICD9-CM code ‘401’ (EH). It is important to remark the absence of the codes ‘250’ and ‘401’ for some diabetic, and hypertensive patients, respectively. Furthermore, some pathologies are not coded when the purpose of the visit of the patient is not the chronic disease itself. However, the lack of this information can be supplied by pharmacological dispensation provided by the Spanish public health service, since drug payment is automated. Therefore, we can claim that drug information is highly reliable, and is better coded than that related to diagnoses. However, although ATC codes may be more relevant for identifying chronic patients in this regard, diagnosis codes are also important. For example, the code ‘272’ (disorders of lipoid metabolism) is a key feature for discriminating healthy individuals from diabetic and hypertensive patients (see Table 7). Note that hypertension is related to metabolic disorders [39]. Also, the code ‘724’ (other and unspecified disorders of back) may be used to distinguish between hypertensive and diabetic patients, since this is a common problem in diabetics [40]. The diagnosis code ‘496’ (chronic airway obstruction) and the ATC ‘N05AL’ (antipsychotics) appear in all of the classification trees for discriminating patients suffering from DM and/or EH, in conjunction with another chronic condition. Previous works have already shown the association between antipsychotic or mental disorders and chronic conditions [41, 42].

Regarding demographic data (age and gender), this study has shown that they are not the most influential for distinguishing among CRGs. Clinicians did not selected gender in any of the decision trees, whereas age was selected in the deeper nodes of the trees, just to separate between CRG-6144 and CRG-7071, with the oldest patients belonging to CRG-7071. This confirms the association between the number of dominant chronic conditions and age. Furthermore, as a consequence of aging, the prevalence of chronic pathologies is increasing considerably in modern societies.

Finally, it is also important to note that this study has analyzed data collected during a single year, which is a short period of time for extracting long-term characteristic patterns associated with patient health statuses. As future work, we will analyze data from chronic patients across several years, capturing information about their temporal evolution. This may enhance the classifiers’ performance and may help us to extract more knowledge about chronic conditions.

## Conclusion

This paper introduces a methodology for creating visually guided classification trees. The approach uses data visualizations to guide and facilitate the feature selection process at each of the tree nodes in an intuitive and easy-to-comprehend way. On the one hand, by using our methodology, analysts can check whether the found relationships between features and health statuses are in accordance with the current clinical knowledge. On the other hand, analysts can formulate hypotheses about new insights, which should be appropriately validated, for instance, through clinical trials.

## Data Availability

The dataset analyzed during the current study are not publicly available but they could be available from the corresponding author on reasonable request.

## Abbreviations

ATC: Anatomical, Therapeutic, Chemical
CRG: Clinical Risk Group
DM: diabetes mellitus
EH: essential hypertension
EHR: Electronic Health Record
ICD9-CM: International Classification of Diseases, Ninth Revision, Clinical Modification
ICPC: International Classification of Primary Care
LDA: Linear Discriminant Analysis
PCS: Patient Classification System
SC: Star Coordinates
SVM: Support Vector Machine
UHF: University Hospital of Fuenlabrada
